# Kikuchi-Fujimoto Disease: A case report and review of the literature

**DOI:** 10.1186/1757-1626-1-187

**Published:** 2008-09-26

**Authors:** Sonna Ifeacho, Theingi Aung, Mojisola Akinsola

**Affiliations:** 1 Department of General Medicine, Heatherwood Hospital, London Road, Ascot, SL5 8AA, UK

## Abstract

Kikuchi-Fujimoto Disease is a rare benign, condition of necrotising histiocytic lymphadenitis. A case of a 55 year old gentleman is described here. He presented with fevers, weight loss and tender cervical lymph nodes. Kikuchi-Fujimoto disease was diagnosed after cervical lymph node biopsy. Symptomatic treatment was provided and an uneventful full recovery was made. Of significant note, this patient's daughter had Kikuchi-Fujimoto disease almost a decade earlier. Although Kikuchi-Fujimoto disease has been reported widely, we believe this is the first familial report in the literature of a case of Kikuchi-Fujimoto disease occurring in a parent and child.

## Background

The presenting complaint of neck masses in association with non-specific systemic signs and symptoms prompt investigation towards the more common diagnoses. However, rarer conditions must still be considered especially when a patient's condition fails to abate. Here in we discuss a case of male patient who presented with a neck mass that was not attributable to the more common causes.

## Case presentation

A 55 year old gentleman presented with a two week history of fevers, anorexia and weight loss. There were no other complaints. He had been previously fit and well and was on no medication. On examination he was lethargic, but otherwise looked well. He was afebrile and haemodynamically stable. Significant findings were generalised lymphadenopathy, palpable in the occipital, posterior auricular, cervical, axillary and inguinal regions. There was 2 cm smooth, tender hepatomegaly. Blood tests revealed neutropenia, hyponatraemia, raised alkaline phosphatase and C-reactive protein. Electrocardiogram and chest radiographs were normal. His initial management consisted of fluid restriction and regular paracetamol, whilst results of further tests were awaited. These included blood and sputum cultures, autoimmune and viral screen.

Subsequently the axillary and inguinal lymphadenopathy resolved along with reduction in size and tenderness of the cervical lymph nodes. There were persistent intermittent temperature spikes and a two day episode of self-resolving diarrhoea.

Blood, urine and stool cultures were negative. Sputum cultures grew respiratory tract flora and were negative for acid-fast bacilli. Paul Bunnell test was negative. The autoimmune screen was negative, as was toxoplasma and cytomegalovirus screens.

Computerised tomography demonstrated generalised lymphadenopathy in the supraclavicular area; axillae, posterior mediastinum, para-aortic areas and deep in the pelvis and groin. The abdominal viscera were normal. Excisional biopsy of a cervical lymph node confirmed a diagnosis of Kikuchi-Fujimoto disease (KFD). Histological analysis showed histiocytic granulomatous infiltration with widespread necrosis of the lymph node extending beyond its capsule into the surrounding fat. No active treatment was instigated and the patient was discharged home after a two week in-patient stay.

At follow-up he reports no symptoms, remains well and there are no abnormalities on clinical examination.

The patient's daughter had KFD seven years earlier and was a patient in the same hospital. She presented with tender cervical lymphadenopathy of two months duration, associated with two stone weight loss, fevers and night sweats. She had no past medical history of note.

The only finding on examination was left posterior auricular lymphadenopathy. Routine blood tests, blood cultures and an autoimmune screen were negative. Blood tests showed a mild leucopenia. ESR and CRP were mildly raised. She had normal renal and liver function.

A lymph node biopsy taken from the posterior auricular area established a tissue diagnosis of KFD. She improved during her in patient stay, with her bloods returning to normal prior to discharge. At review one week and one month later she had made a full recovery.

## Discussion

KFD is a benign histiocytic necrotising lymphadenitis. KFD is rare, but most common in Asia. In the early 1970s both Kikuchi and Fujimoto first described cases of KFD in Japan [1,2]. Its aetiology has not yet been fully determined, however it is believed it may be of viral origin, EBV, HHV6 and 8 have been suggested. Raw fish was postulated as a cause, but the recent literature doesn't support this [3]. An autoimmune aetiology is also likely as it has been reported in association with SLE. It tends to affect a young population under 30 years of age, including children, although the latter are less commonly affected. There are reported cases in an older age group and pregnant women too [4]. Early reports suggest affected female cases are more common; however more recently this view has changed to one of equal prevalence in both genders.

The most common signs and symptoms are lymphadenopathy, fever, sweats, malaise, anorexia, weight loss, hepatomegaly and leucopenia [3]. The viral aetiology of KFD is supported by its non-specific self-resolving symptoms, which are of slow, insidious onset.

A definite diagnosis is made by tissue biopsy, indeed whole lymph node biopsy. Histopathological assessment of affected lymph nodes reveals characteristic findings. There are three main patterns identified, proliferative, necrotizing and xanthomatous. The proliferative picture is seen in approximately a third of cases and has a dominant inflammatory infiltrate. Half of cases show necrotizing pattern and the xanthomatous type is rare and has abundant foam cells [5]. Immunoblast cell changes seen in lymph nodes mimic those of malignancy and are a source of diagnostic confusion. Cellular protein structures have been noted in the cytoplasm of lymphocytes and histiocytes that have also been found in those cells of patients with SLE. This adds strength to the hypothesis that KFD is a self-limiting SLE-like disorder.

As the symptoms are non-specific and some of the histological features are similar to other diseases it is easy to misdiagnose KFD with SLE or lymphoma. This is important as the treatment of KFD is symptomatic and supportive, spontaneous recovery is usual, while the latter two conditions require prompt specific treatments.

Long term follow-up of these patients is necessary as recurrent cases of KFD have been reported and there is some belief that KFD may be a precursor for SLE, as both diseases have had concurrent and co-existing disease patterns in the same patients [6-8]. In a review of KFD cases by Kucukardali et al the reported overall mortality rate associated with KFD is 2.1% [9].

## Conclusion

KFD is uncommon, but should feature in a list of differential diagnoses of tender lymphadenopathy, especially affecting the cervical region. Its treatment differs significantly from the other conditions that would be on that list such as SLE, lymphoma and TB. Lymph node biopsy will aid accurate diagnosis, but if confusion with SLE occurs differentiation can be made with the aid of blood tests for complement levels amongst others.

Recurrence has been reported. KFD has been reported within a family, but not between parent and child. This case is believed to be the first reported one. It may suggest a genetic predisposition or a common environmental factor. Long term follow up will give us more data.

## Learning points

• KFD is an important diagnosis to make as treatment is conservative and this differs to the management of other conditions it may be confused with.

• KFD requires a tissue diagnosis and therefore lymph node biopsy should be considered in patients with cervical lymphadenopathy and systemic upset.

• When clinical features suggest a diagnosis of KFD, a family history should be sought.

## Competing interests

The authors declare that they have no competing interests.

## Authors' contributions

All authors read and approved the final manuscript.

## Consent

Written informed consent was obtained from the patient for publication of this case report and any accompanying images. A copy of the written consent is available for review by the Editor-in-Chief of this journal.
